# Facial expression analysis in a wild sporting environment

**DOI:** 10.1007/s11042-022-13654-w

**Published:** 2022-09-15

**Authors:** Oliverio J. Santana, David Freire-Obregón, Daniel Hernández-Sosa, Javier Lorenzo-Navarro, Elena Sánchez-Nielsen, Modesto Castrillón-Santana

**Affiliations:** 1 Universidad de Las Palmas de Gran Canaria, Las Palmas, Canary Islands Spain; 2 Universidad de La Laguna, San Cristóbal de La Laguna, Canary Islands Spain

**Keywords:** Soft biometrics, Expression recognition, In the wild

## Abstract

The scientific community and mass media have already reported the use of nonverbal behavior analysis in sports for athletes’ performance. Their conclusions stated that certain emotional expressions are linked to athlete’s performance, or even that psychological strategies serve to improve endurance performance. This paper examines the portrayal of well-known emotions and their relationship to the participants of an ultra-distance race in a high-stake environment. For this purpose, we analyzed almost 600 runners captured when they passed through a set of locations placed along the race track. We have observed a correlation between the runners’ facial expressions and their performance along the track. Moreover, we have analyzed Action Unit activations and aligned our findings with the state-of-the-art psychological baseline.

## Introduction

Emotion is a central feature in many sporting events. Athletes can experience many feelings, including anger, disgust, fear, happiness, sadness, and surprise. Most emotion theorists argue that emotions have the power to motivate and regulate cognitions and behaviors in team and individual sport performance [5, 18] as it provides information about what is going inside an athlete [14]. Over recent years, some authors have highlighted that emotional expressions are factors that can affect opponents in team sports and persuade them of their better abilities [16, 18]. Consequently, different research works have examined how emotional expressions directly influence the set of actions carried out by opponents in team sports [6, 18, 27]. Other works have highlighted how certain psychological strategies, such as often smiling, are used by elite athletes to reduce their awareness of effort sensations during endurance running [5]. However, research on emotional expressions resulting from physical effort is a recent field that is related to the extraction of health cues from biometric data [30]. To date, uniquely a few conducted experiments have begun studying participants’ responses to exercise-related stimuli with a positive or negative facial expression only at the beginning of the activity but not during its performance [4, 5].

**Fig. 1:**
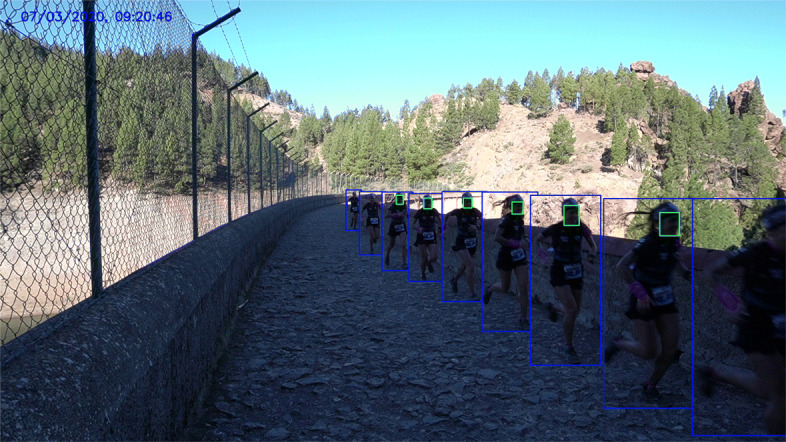
Collage of a subset of the samples used for a runner in a recording point. Blue and green containers correspond to body detections that include a valid face detection

In this context, our work aims to take a step forward in researching emotional expressions in the wild, considering an individual activity environment with participants from an ultra-distance race, where the relationship between facial expression and physical performance is analyzed. Unlike a football pitch or a tennis court [18], ultra-distance races suppose a more challenging scenario for computer vision systems due to the diversity of issues present, i. e., varied illumination conditions, facial occlusions, blur images due to motion, and low facial resolution images. In addition, the low-resolution problem frequently causes the performance degradation of facial expression recognition methods under real-life environments [38]. Figure 1 shows a composition with a set of samples for an individual in a particular recording point to provide the reader a better comprehension of the available information per runner.

As a result, this work advances toward finding patterns in ultra-distance runners captured in a real-life competition under different environmental conditions. Combining state-of-the-art facial expression recognition techniques and tracking algorithms, we analyze the facial expression of the runners at different points along the race track and evaluate the emotional trends over the distance covered and the time spent. Our findings show some regular patterns, often emotions related to fatigue and physical tension, which can be correlated to the track conditions and the runner’s performance.

Besides emotions, facial recognition systems can track the movements of specific facial muscles. As a case study, we focus on the zygomatic major muscle, whose activation is strongly related to physical effort in athletes according to Uchida et al. [35]. We measure the activation frequency of this muscle in the runners to assess the level of fatigue at different points of the race. The activation frequency and runner’s effort (based on a race profile analysis) align with Uchida et al. conclusions. In addition, we compare these results with the rankings of previous race editions to contextualize them according to individual runner performance and expectations.

The paper is organized as follows. Section 2 discusses previous related work with particular emphasis on the state-of-the-art facial expression recognition systems and the main characteristics of biometrics in running competitions. Section 3 describes the runner image dataset used to perform our experiments. Section 4 describes our proposal for a facial expression recognition pipeline. Section 5 details our experiments and results. Finally, Section 6 provides our concluding remarks.

## Related work

Wilhelm Wund’s seminal work developed a structural description of subjective feelings states based on a three-dimensional space [37]. Wundt suggested that each feeling state could be defined according to its positioning along three axes: valence (positive vs. negative), arousal (active/aroused vs. passive/calm), and tension (tense vs. relaxed). Since the latter would imply a hard-to-measure aspect of human feelings, it is usual to consider only the valence and arousal dimensions, mapping the feeling states in a circle around these two axes [31].

In order to ground this two-dimensional map, Scherer introduced appraisal dimensions, which had a substantial impact on emotion differentiation [32]. These appraisal dimensions were goal conduciveness (conducive vs. obstructive) and coping potential (high power/control vs. low power/control).

Subsequently, in the early ’70s, Ekman and Friesen conducted a cross-cultural experiment that defined six basic emotions in addition to the neutral face: anger, disgust, fear, happiness, sadness, and surprise. Hence, these authors confirmed that humans perceive specific basic emotions in the same way regardless of culture [10]. Moreover, Ekman and Friesen created the Facial Action Coding System (FACS). It is a comprehensive, anatomically based system for describing all visually discernible facial movements. It breaks down facial expressions into individual components of muscle movement, called Action Units (AUs). Consequently, multiple facial expression recognition (FER) systems have been explored to encode expression information from facial representations.

### Facial expression recognition systems

Over the past decade, the research on automatic facial expression analysis has been broad in recognition techniques due to the development of numerous applications in sociable robots, medical treatment, and other human-computer interaction systems. According to the data representation, a recent survey [22] classifies FER systems into two main categories: static image FER and dynamic sequence FER. In static-based methods, the feature representation is only encoded with spatial information from the current single image [23, 26]. In contrast, dynamics-based methods also consider the temporal relation between contiguous frames in the input sequence [12, 19].

Recently, several deep learning architectures for FER have emerged. Bodapati et al. [3] proposed a deep convolutional neural network model (FerNet). This model comprises a sequence of blocks consisting of multiple convolutional and sub-sampling layers. Similarly, Pham et al. [29] proposed a deep neural network architecture with the attention mechanism (ResMaskNet). These authors used a so-called masking idea to boost CNN performance in the facial expression task. It uses a segmentation network to refine feature maps, focusing on relevant information to make correct decisions. Both architectures FerNet and ResMaskNet, were trained on the FER2013 dataset [15]. This collection contains 35.887 grayscale images (48x48), from which 28.709 were used for training, 3.589 for validation, and 3.589 for testing. Each image is collected from the Internet and labeled as one of the seven categories: anger, disgust, fear, happiness, sadness, surprise, and neutral. However, the dataset contains several wrong samples (e.g., non-face images or faces cropped incorrectly), and the image distribution among emotions is highly unbalanced. For instance, 8.989 faces elicit happiness, whereas 547 faces elicit disgust.

Several toolboxes have helped to disseminate some of the previously described techniques widely. The Python Facial Expression Analysis Toolbox (Py-Feat) [7] is an open-source package that supports facial expression data analysis. It provides tools to extract facial features like OpenFace [1] and modules for preprocessing, analyzing, and visualizing facial expression data. Moreover, this toolbox provides four different FER techniques: Random Forest (RF), Linear Support Vector Machine (SVM), FerNet, and ResMaskNet. The RF and the Linear SVM were trained on HOG extracted from ExpW, CK+, and JAFFE datasets [39]. They applied 3-fold cross-validation for identifying the best hyperparameters. Serengil and Ozpinar developed another relevant toolbox known as Deepface [33]. This framework provides face recognition and facial attribute analysis (age, gender, emotion, and race). Their FER model involves three convolutional blocks and two fully connected layers for a 48x48 grayscale input, which was trained with the dataset created for a Kaggle Facial Expression Recognition Challenge^1^.

### Biometrics in running competitions

In the recent past, biometrics has been broadly applied to individual and team sports to improve performance [21, 25, 34]. However, the application of biometrics to recognize or describe individuals for any purpose is still not so frequent. Even the athlete recognition problem has evidenced the difficulties in the sporting scenario. Indeed, most literature is devoted to identifying runners solving the Racing Bib Number (RBN) recognition problem [2, 17]. The advantage of this approach is that there is no previous identity registration problem. However, the first drawback is that the bib number may be occluded. The main concern is that it is evident that there is no absolute identity recognition, as the bib number may be exchanged during the competition. Even the original identity associated with the bib number may be faked during the competition.

To overcome the possibility of bib number occlusion, some authors have combined face and bib number cues to improve overall performance [36]. Other authors have explored the body appearance to re-identify individuals in different locations, separated up to 20 hours, based on the body appearance [28]. Their results suggest a poor performance of standard re-identification techniques due to severe illumination changes, scenario changes, clothes changes, and even similar garments (people from the same club/team)

Contrary to the previously described approach, the gait trait has also been adopted recently [8]. These authors used the arm swing features extracted from the silhouette to remove the problems present in RBN, face occlusion, or the similarity in clothing appearance.

Although not for the running scenario, a commercial automatic FER is adopted by Hopfensitz et al. [18] to assess football teams’ performance after the detection of anger and happiness in the players’ portraits captured during 40 years of the FIFA World Cup. Their conclusions suggested a correlation between anger with allowing fewer goals, and happiness with scoring more goals.

Recently, from a business viewpoint, the website MarketandMarkets, has highlighted the importance of driving market growth with the support of FER systems, the adoption of the Internet of Things (IoT), and the evolution of deep learning technologies in order to grow approximately USD 17.6 billion by 2026 [24]. However, automatic FER has not been considered in the running context to the best of our knowledge. Nonetheless, psychological studies have addressed the adoption of cognition strategies in athletics, training runners to display particular facial expressions, to reduce the effort sensations in endurance competitions, i.e., for running economy [5]. Therefore, we consider promising to explore the features of FER in sporting environments to analyze the performance of endurance athletes, and then validate the proposed approach to show the findings obtained in practice with runners in wild sporting environments.

## Dataset

A real competition dataset used to explore biometrics in the ultra-distance runners scenario was gathered during Transgrancanaria (TGC) 2020, held in Gran Canaria on March 2020. Among the different TGC tracks, covering each one different distance, we focused on the Classic 128km trail race that, as suggested by the name, challenges the participants to cover 128km on foot in less than 30 hours. With that objective in mind, the whole group of participants departed on March 6th, 2020, at 11 pm, reaching the fastest runners at the finish line roughly 13 hours later.

For the 2020 edition, there was a lower number of participants than in previous editions due to the initial state of the COVID-19 pandemic. Therefore, of the original 900 signed-up participants, just 677 of them started. Among them, just 435 reached the finish line in time. A relevant subset of runners was recorded across the track using Sony Alpha ILCE 6400 cameras with a 16-50mm lens and configured at 1920x1080@50fps.

**Fig. 2:**
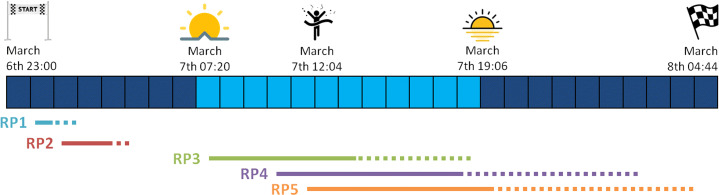
TGC Classic timeline for the 2020 edition, from the race start until the last runner’s arrival. The lines below the timeline indicate the time runners were passing through a given RP. The continuous part of each line is the recorded portion. The winners needed just 13 hours to complete the race

Figure 2 illustrates the progress of the race over the 30 hours it lasted. The reader may observe that given the starting time, eight hours before sunrise, participants were recorded first with nightlight conditions. Table 1 summarizes, for each Recording Point (RP), the distance from the starting line, the starting recording time, and the number of identities annotated at each RP. In the first two RPs, runners arrived in groups and occlusions were frequent, especially at RP1. The runners were more widely dispersed in the rest of the RPs, reducing the number of occlusions. It can be observed that the number of annotated runners is decreasing from RP2 onwards; the closer to the finish line, the more significant the gap between the leaders and the last runners, making the recording task longer for the exhausted recording team (composed basically by the authors).

**Table 1:** Dataset statistics

	Km	Start Rec. Time	# annot. runners
RP1	16.5	00:14	419
RP2	27.9	01:19	586
RP3	84.2	07:40	203
RP4	110.4	10:30	139
RP5	124.4	11:44	114

**Fig. 3:**
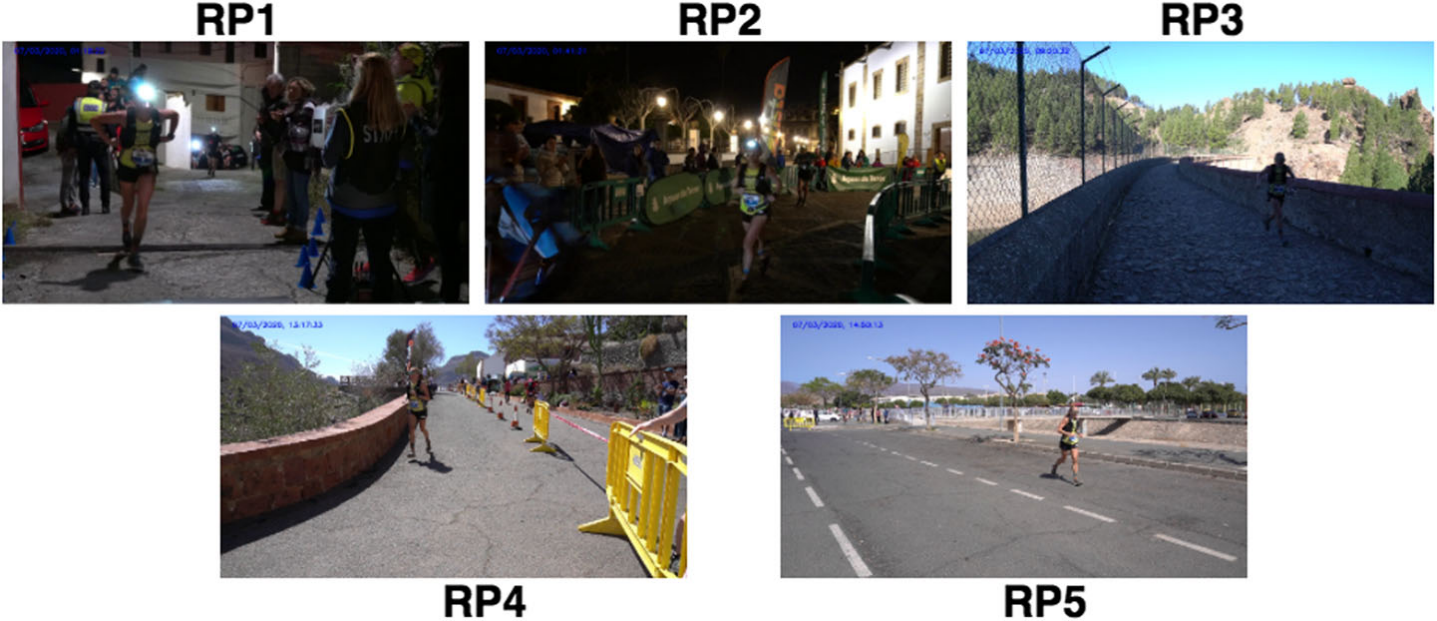
A runner captured at each RP. The reader may observe the challenging conditions to capture the face, particularly in RP1, and the different illumination and resolutions across RPs

Figure 3 depicts images for the same individual in the different RPs, allowing the reader to compare the dataset scenarios. The first two RPs are located in the first 30 km; those images are collected with nightlight. The context of RP1 is particularly challenging for face detectors, as reported below, because runners have mostly the headlamp on, as they have just left a dark path. The original footage comprises a video recording at 50fps. We have used that video stream but just considered 25fps to reduce the number of frames for our purpose.

## Face processing pipeline

**Fig. 4:**
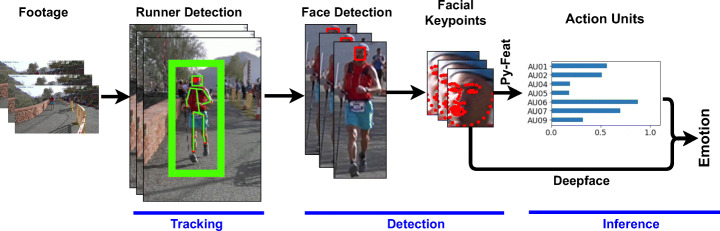
Proposed pipeline. Runners are detected using YOLO and tracked with ByteTrack. Also, skeletons detected with AlphaPose are available. The skeleton with the more prominent intersection with the runner bounding box is adopted to restrict accurate face detection

This work proposes and evaluates a sequential pipeline divided into three main steps, namely tracking, detection, and inference (see Fig. 4). The tracking step consists of a robust people tracker, ByteTrack [40], which reports several tracklets of runners, staff, and the public. The tracker is applied using an annotated bounding box, which comprises information about the source frame and the capture timestamp. The runner’s identity is manually annotated based on our previous research works [13, 28] after applying pedestrian detection at 1fps. Additional runner samples are computed by processing up to seven seconds around the annotated runner bounding box. After applying the tracker approach, the tracklet that matches an annotated identity is adopted as the runner tracklet at each RP. The race scenario is challenging given the runners can be occluded by other runners, race, staff, or the public, and therefore, in case of temporary occlusions. ByteTrack applies a tracklet interpolation that effectively obtains the boxes of occluded objects. Thus, given a tracklet *T* which box has been lost due to occlusion between frame *f*1 and frame *f*2, then the interpolated box of tracklet T at frame t is computed as follows:

1 $$ B_{f} = B_{f_{1}} + (B_{f_{2}} - B_{f_{1}})\frac{f - f_{1}}{f_{2} - f_{1}} $$

Where *B*_*fi*_ represents the tracklet box of *T* at a frame *f*_*i*_ and *f*_1_ < *f* < *f*_2_. Zhang et al. reported that the interpolation best performance occurs when *f*_2_ − *f*_1_ ≤ 20 [40].

In addition, body skeletons in the frame are estimated using AlphaPose [11], serving to filter for each runner situation with multiple face detections. In those cases, the runner face detection must contain the skeleton head center.

Once the runners are detected, we perform the face detection step over the body images. Given the wild characteristics of the problem context, it is certainly not well suited for most face detectors. The original dataset described in the previous section, subsampled at 1fps, was adopted to assess the performance of these algorithms. Among the available detectors, we have evaluated three of them: the dlib implementation of Kazemi and Sullivan [20] denoted below as DLIBHOG, MTCNN [41], and RetinaFace [9]. The wild scenario made DLIBHOG and MTCNN reported a TPR lower than 15%, while RetinaFace obtained a TPR over 41% (see Table 2). Consequently, we have selected RetinaFace as our face detector. Figure 1 includes a set of detections in RP3 using RetinaFace, after filtering face detections using the runner skeleton.

**Table 2:** Face detection results in terms of true positive rate (TPR) for several annotated runners’ bounding boxes (BBs)

				RetinaFace
RP	# imgs	# runner BBs	# TP	TPR
RP1	1172	1589	298	0.19
RP2	1234	1445	657	0.45
RP3	618	526	319	0.61
RP4	281	255	213	0.84
RP5	250	253	189	0.75
Total	3555	4068	1676	0.41

The number of true positives (TP) is also provided

Once the face detection step has been performed, the inference step is applied to acquire the relevant facial emotion information. With this aim, we have adopted a set of off-the-shelf classifiers integrated into the Deepface [33] and Py-Feat [7] frameworks. The former provides a CNN model trained with 48x48 pixel samples. In contrast, the latter provides four models, two handcrafted models, random forest (RF) and support-vector machine (SVM), and two deep learning models, FerNet [3] and ResMaskNet [29].

**Fig. 5:**
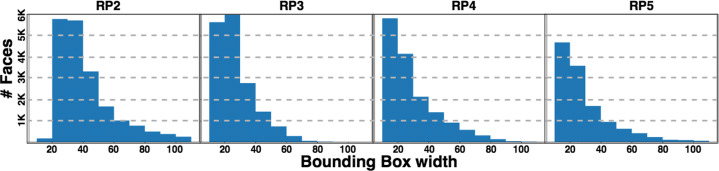
Facial dimensions distribution across RPs

Since our work is focused on a wild environment, the recording conditions are far from ideal because the weather, the illumination, and the camera location affects the number of valid faces and their dimensions. Figure 5 summarizes the distribution of detected faces according to their width. Although the smallest faces used to train any adopted models are 48 pixels wide, most detected faces are under these dimensions. In order to avoid artifacts due to low resolution upscaling, our experiments only use faces having a width of 40 or more pixels. Table 3 summarizes the number of faces detected in the available body images, also indicating the number of detected faces reaching the minimum size.

**Table 3:** Body and face detection statistics

	# bodies	# faces	# faces ≥ 40px
RP2	41492	20526	8854
RP3	32346	17186	2536
RP4	19878	15822	3379
RP5	173189	12552	2500

The challenging nightlight conditions should also be taken into account in RP1 and RP2, where the runner images were captured in the middle of the night (see Table 1). Indeed, nightlight significantly decreased RetinaFace performance in RP1, since many runners wear their headlamps on. Even if the number of runners and corresponding body bounding boxes is higher than in other RPs, the number of detected faces is lower. Because of this poor facial detection, and considering that runners were at the beginning of their challenge, we excluded RP1 from the experiments reported in the next section.

## Experiments and results

The chosen FER approaches assign an emotion to each processed face according to seven categories, that is, the neutral state and the six basic emotions: anger, disgust, fear, happiness, sadness, and surprise. Since this set of emotions does not consider facial expressions that might be expected from ultra-distance runners, such as tension or fatigue, it becomes necessary to identify these expressions for correctly interpreting our classification process results. However, there is no scientific and objective method to quantify and compare emotions, thus making it necessary to rely on qualitative analysis. For this purpose, we have analyzed the obtained facials expression from different perspectives aiming to use a valid baseline.

**Fig. 6:**
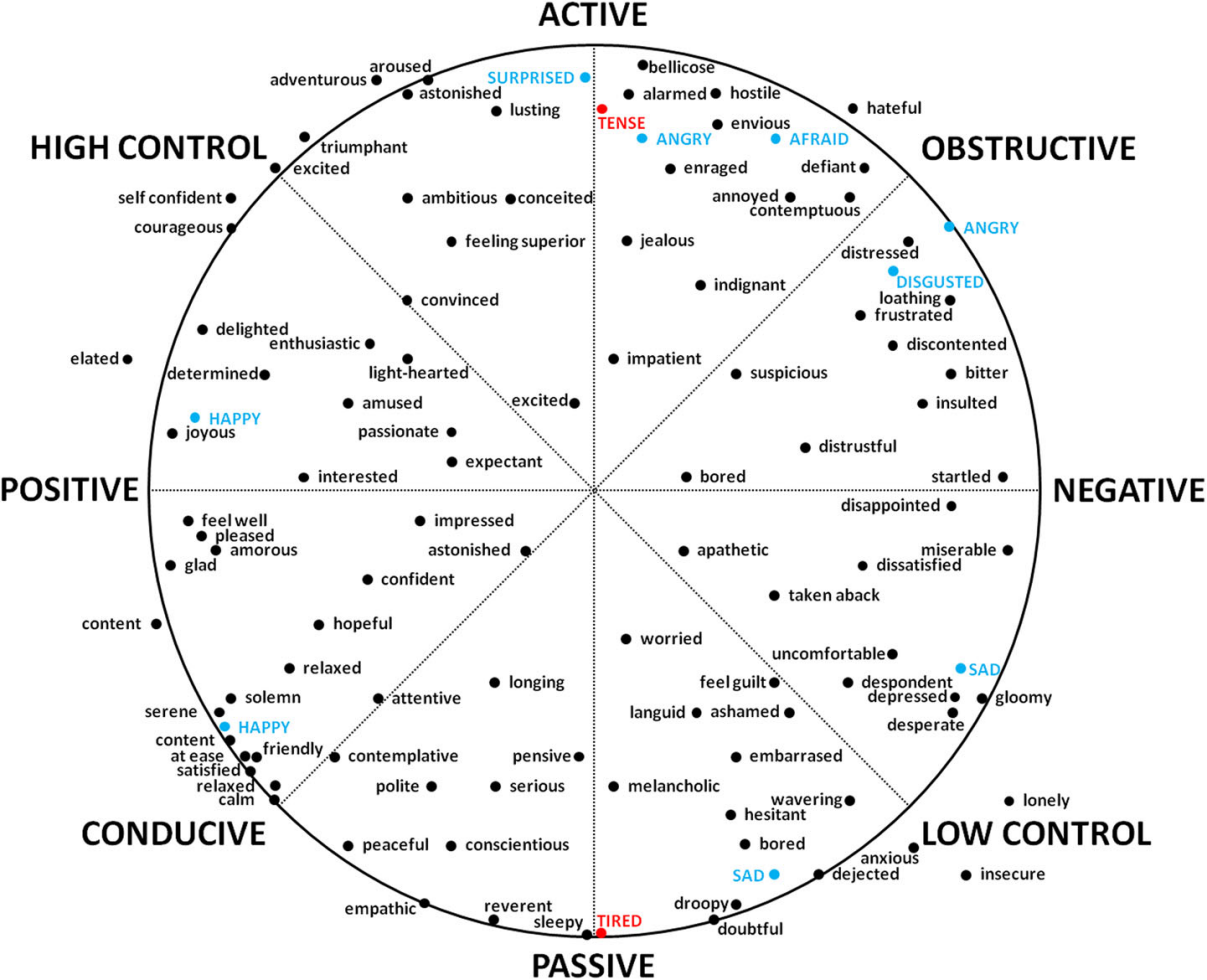
Emotion mapping in a two-dimensional space. The horizontal axis shows emotion valence, the vertical axis shows emotion arousal, the main diagonal shows coping potential, and the antidiagonal shows goal conduciveness

Figure 6 shows an illustration of Scherer’s circumplex model [32] in which we have highlighted the six emotions detected by the adopted classifiers (blue) and the two emotions that we expect to be more frequent in ultra-distance runners: tense and tired (red).

According to this mapping, an expression denoting physical effort or tension will exhibit high activity and power level. Although an expression of tension cannot be linked, per se, to a positive or negative valence state, it could be considered as reflecting an obstructive view of goal attainment because the runner is struggling to overcome a challenge. Emotions with similar qualities are anger, surprise, and fear.

On the other hand, a tired expression showing physical fatigue or exhaustion will reveal a low activity and power level. A tired expression cannot be linked to a positive or negative valence state either. However, instead of exhibiting an obstructive view of goal attainment, it will show quite the opposite, as the runner will no longer be focused on the challenge due to fatigue. The closest emotion to these traits is sadness.

**Fig. 7:**
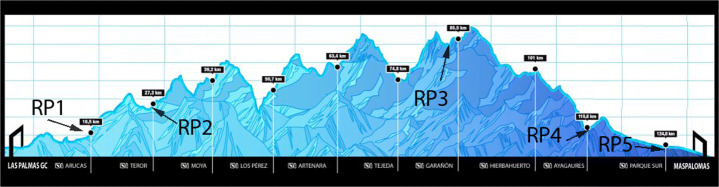
TGC Classic track profile (adapted from source image courtesy of Arista Eventos SLU)

Therefore, concerning the six emotions detected by our classifiers, we expect sadness, anger, surprise, and fear to stand out. We do not expect significant values for disgust or happiness. Figure 7 shows the TGC Classic profile along with an indication of where each RP was located. We use this profile to interpret the results provided for the evaluated FER models at the four analyzed RPs (RP2 to RP5). Sections 5.1, 5.2, and 5.3 discuss the existent correlation between the obtained emotions and the participant’s performance from a generic point of view, whilst Sections 5.4 and 5.5 go deeper on how fatigue is detected through a specific AU activation.

### Overall FER results

Given a runner’s face, each model estimates the conditional probability of each expression (neutral state plus six basic emotions). These estimations represent the confidence on the corresponding emotion. They are percentage values, so the sum of the seven values equals 100. Once all estimations are computed, the higher-rank emotion is assigned to the face.

We extend this same procedure to assign an emotion to a particular runner. First, we apply the model to compute the score of each runner’s available faces along the sequence of frames of the captured clip. Next, we add the score for each category and choose the emotion corresponding to the category with the highest aggregate score.

**Fig. 8:**
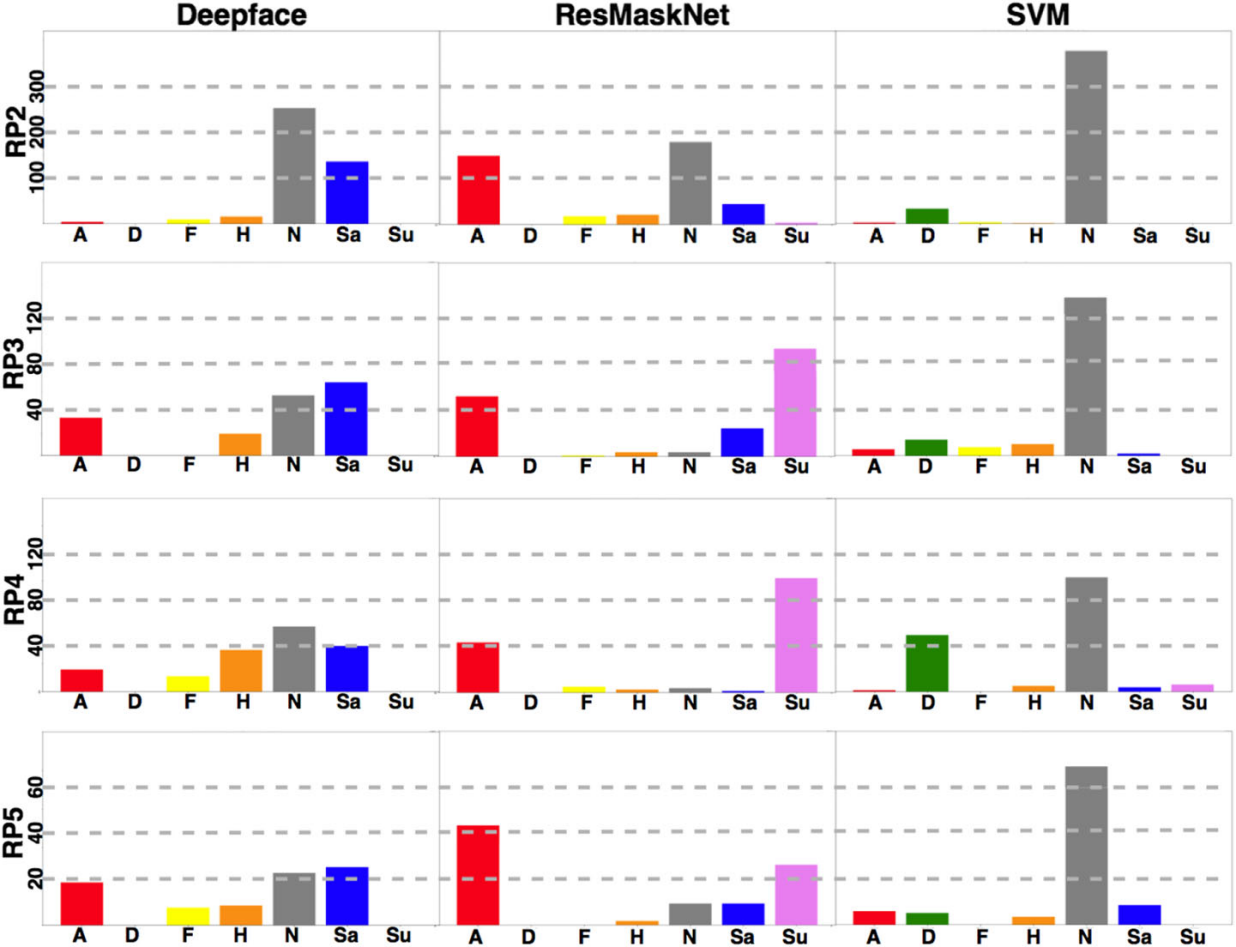
Total number of runners displaying a particular emotion in each RP. We consider the neutral state (N) and the six basic emotions: Anger (A), Disgust (D), Fear (F), Happiness (H), Sadness (Sa), and Surprise (Su)

**Fig. 9:**
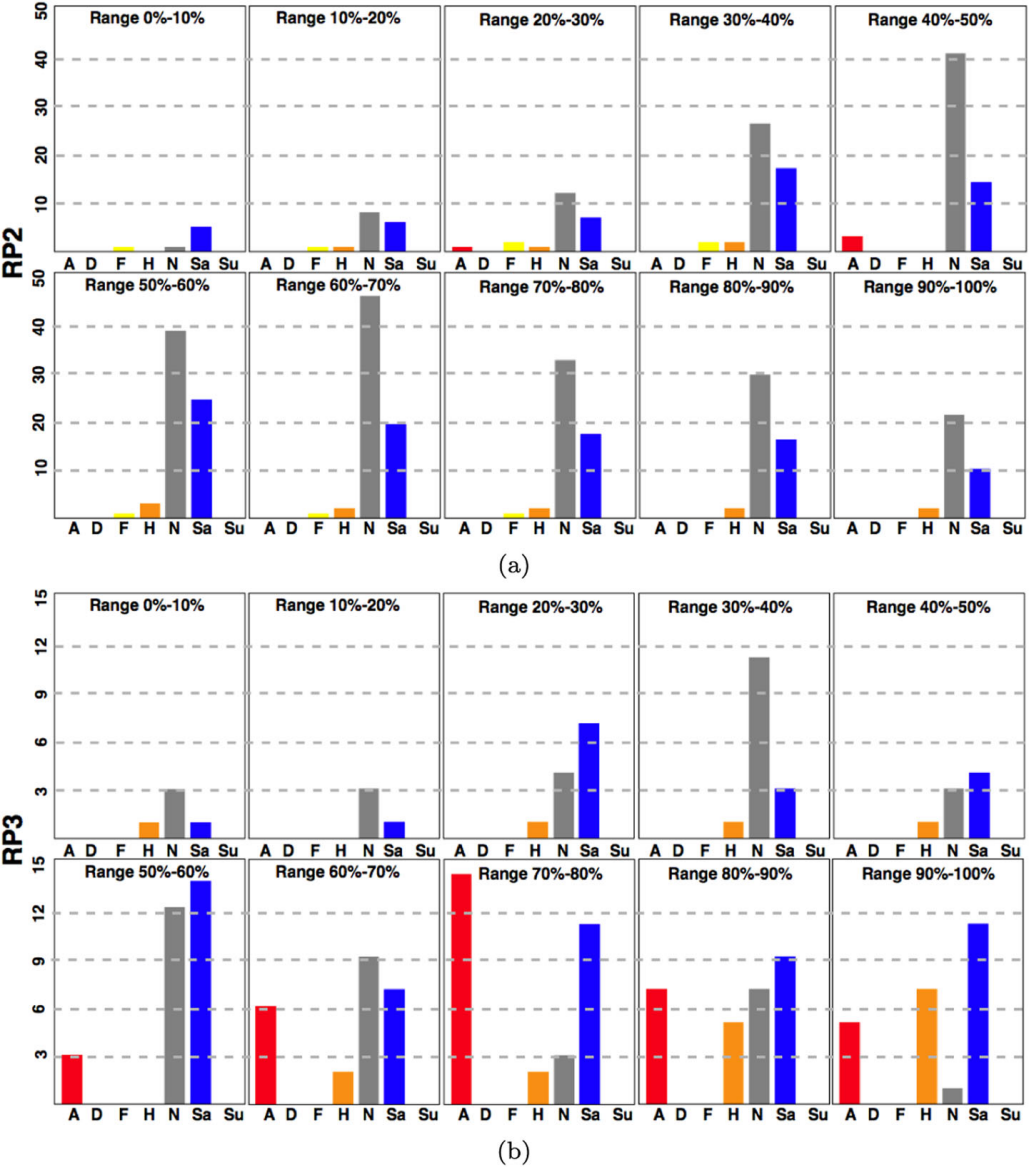

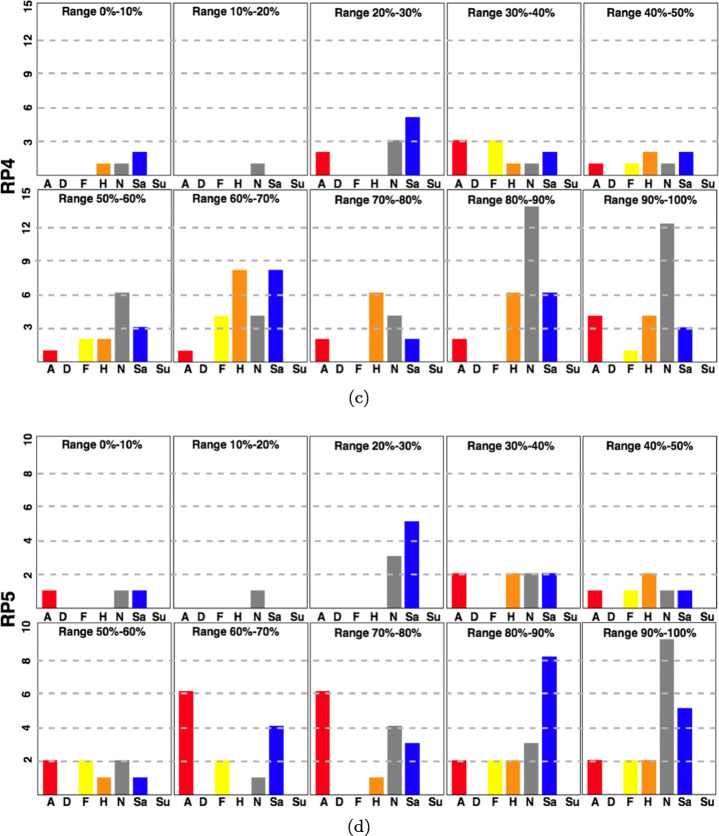
Total number of runners displaying a particular emotion for RP2 and RP3 according to the Deepface model. Data are divided chronologically by deciles. We consider the neutral state (N) and the six basic emotions: Anger (A), Disgust (D), Fear (F), Happiness (H), Sadness (Sa), and Surprise (Su)

Figure 8 compares the results of three FER models in the different RPs. The first one is Deepface, and the other two belong to the Py-Feat toolbox: ResMaskNet and SVM. The RF and FerNet models provided by the Py-Feat toolbox are not included due to their low variability results: RF mainly reported neutral expressions, while FerNet classified most expressions as sad (Fig. 9).

Each bar in Fig. 8 plots the total number of runners captured in that RP showing a particular emotion.

The results for the three models shown in a particular RP are comparable, since the total number of analyzed runners is the same. However, it should be considered that the number of runners varies among different RPs. Hence we have decided to show absolute numbers instead of percentages.

The results obtained show that the Deepface model classifies most runners as having a neutral or sad expression. Neutral expressions are a vast majority in RP2, where runners had only traveled 30km. The difference between both expressions becomes smaller as runners cover more distance. In fact, sad expressions outnumber neutral expressions by a wide margin in RP3. As shown in Fig. 7, RP3 is located about 80km from the starting point, 1km before the central water station. The runners reach that RP after covering most of the uphill part of the track and a whole night of physical effort, so it is natural that the emotion closer to fatigue abounds. Another common emotion in the RPs located further away from the starting line is anger, a sign of runners’ physical strain after running such a long distance.

Anger and surprise are the two emotions most frequently detected by ResMaskNet. These emotions are related to the facial expressions of fatigue, so this is a consistent outcome. The high number of neutral expressions in RP2 is an outlier result for this model, although it could be justified by the fact that runners have traveled a shorter distance when they reach this RP and that they must run all night before reaching the next one.

SVM is the least sensitive of the three models and reports that most facial expressions are neutral. This outcome provides a clear example of how difficult it is to identify emotions in the wild, as we can find poorly illuminated faces as small as 40 pixels in width, whereas the model was trained with 200-pixel width faces. Some unexpected results from RP4 highlight these difficulties, as Deepface reports a significant amount of happy expressions, while SVM identifies a remarkable number of disgust expressions. Therefore, even though most of the results are consistent, it is nevertheless true that emotion classifiers have a long way to go before they can be deployed in such demanding environments.

### FER comparison

In order to provide more insight into these results, Table 4 compares the emotions identified by the two most sensitive models studied: Deepface and ResMaskNet. There are substantial differences between both models, which indicates the significant challenges posed by the problem we are discussing. The two models agree the most in RP2, but the agreement rate is just 30.6%. They mainly agree on identifying neutral faces, while the primary source of differences is a large group of faces that ResMaskNet identifies as angry and Deepface identifies as neutral or sad to a lesser extent

**Table 4:** Comparison between the emotions provided by Deepface and ResMaskNet

		Deepface					
		N	A	F	H	Sa	Su
		(a) RP2					
ResMaskNet	N	25.1	0.5	0.7	1.4	15.4	0.0
	A	24.8	0.5	0.7	1.0	8.9	0.0
	F	2.7	0.0	0.2	0.0	1.4	0.0
	H	1.4	0.0	0.5	0.7	2.4	0.0
	Sa	6.0	0.0	0.0	0.5	4.1	0.0
	Su	0.7	0.0	0.0	0.0	0.2	0.0
		(b) RP3					
	N	1.7	0.0	0.0	0.0	0.6	0.0
	A	9.5	6.7	0.0	3.9	11.2	0.0
	F	0.6	0.0	0.0	0.0	0.0	0.0
	H	0.6	0.6	0.0	1.1	0.0	0.0
	Sa	6.7	2.2	0.0	0.0	5.6	0.0
	Su	12.3	10.1	0.0	6.1	20.7	0.0
		(c) RP4					
ResMaskNet	N	1.5	0.0	0.0	0.7	0.0	0.0
	A	9.5	2.2	1.5	6.6	6.6	0.0
	F	0.7	1.5	0.0	0.7	0.0	0.0
	H	0.7	0.0	0.0	0.7	0.0	0.0
	Sa	0.0	0.0	0.0	0.0	0.7	0.0
	Su	21.9	8.0	6.6	13.1	16.8	0.0
		(c) RP5					
	N	4.1	1.0	3.1	0.0	3.1	0.0
	A	11.2	13.3	2.0	4.1	13.3	0.0
	F	0.0	0.0	0.0	0.0	0.0	0.0
	H	1.0	1.0	0.0	0.0	0.0	0.0
	Sa	3.1	2.0	1.0	1.0	4.1	0.0
	Su	8.2	5.1	3.1	5.1	10.2	0.0

Results are shown as a percentage of the total number of faces for each RP. We consider the neutral state (N) and five basic emotions: Anger (A), Fear (F), Happiness (H), Sadness (Sa), and Surprise (Su). These classifiers did not identify any Disgust expression

**Fig. 10:**
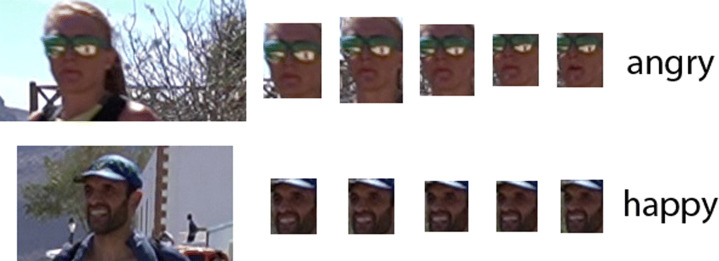
Cropped heads and faces detected by RetinaFace in the preceding five frames for two runners whose expression recognition using Deepface was respectively angry and happy

The agreement rate for the rest of the RPs is significantly lower. The two models only agree on 15.1% of the faces in RP3 and 5.1% of the faces in RP4. Most of the differences lie in the faces that ResMaskNet identifies as angry and, especially, surprised. The agreement rate is higher in RP5, at 21.5%, mainly due to the agreement on many angry faces, although there are many other angry faces on which the models disagree. The expressions identified by ResMaskNet as surprised constitute a significant source of discrepancies since Deepface did not identify any surprised expressions (Fig. 10).

**Fig. 11:**
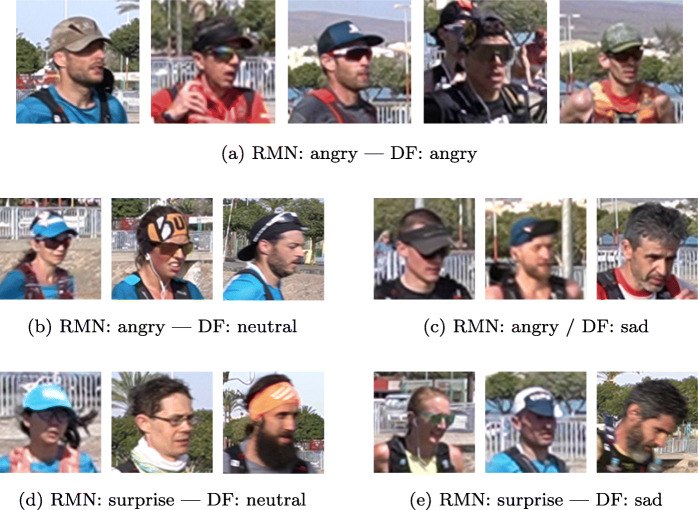
Faces of some selected runners in RP5. Faces are grouped according to the facial expression detected by ResMaskNet (RMN) and Deepface (DF)

Figure 11 shows a sample of faces chosen from those runners captured in RP5. The highest agreement rate between the two classifiers in this RP appears in the angry faces. Figure 11.a shows some examples with expressions of physical tension that could easily be mistaken for expressions of anger by a classifier not trained to showcase the difference between them.

ResMaskNet classifies most RP5 faces as angry, while Deepface provides a larger variety. Figure 11.b shows faces that ResMaskNet classified as angry and Deepface as neutral. Two faces have relaxed expressions that a human observer might identify as neutral. The third has its mouth open and teeth clenched, but the facial musculature is not tense and does not transmit a sense of anger. Figure 11.c shows faces that ResMaskNet classified as angry and Deepface as sad. Certainly, the expression of fatigue suggests an emotion closer to sadness than anger.

As mentioned above, Deepface did not detect any surprised face, while this expression is quite frequent between ResMaskNet proposals. Figure 11.d shows faces that ResMaskNet identified as surprised and Deepface as neutral. It becomes clear that a human observer hardly identifies these faces as surprised. Figure 11.e shows faces that ResMaskNet identified as surprised and Deepface as sad. Again, sadness seems the most appropriate expression for these faces, reflecting great physical exhaustion after a long effort.

### FER results per decile

The preceding sections have shown the general trend at each RP by discussing the total number of runners showing each emotion. However, it should also be kept in mind that the performance of every runner will affect their facial expression. Therefore, we have divided the recording time for each RP into deciles: the first decile starts with the passage of the first runner and the last decile ends with the passage of the last annotated runner. Since Deepface has proven to be the most sensitive model, we have focused on this model’s classification. Figure 9 shows, for each decile at each RP, the total number of runners showing each facial expression according to the Deepface model.

Considering the distribution over time of the expressions provides us more context. For instance, although neutral expressions are the dominant ones in RP2, we can note that the most frequent expression among the fastest runners is sadness, a symptom of the fatigue they experience from making a more substantial effort than the other runners during the first part of the race.

The picture is different in RP3: runners have almost finished the tough uphill and reach the central water station where they may rest and eat. For the first half group, neutral and sad expressions dominate, with some happy individuals. The runners passing during the first half of the recorded time shows similar behavior to the one found in RP2, with neutral and sad expressions dominating. However, during the second half of the recorded time, many angry expressions appear, indicating that the runners have been shifting from fatigue to an expression of tension typical of runners who are experiencing difficulties in continuing in the race.

The results in RP4 deviate from what was expected, as discussed above, and there is a greater variability of expressions. Neutral and sad expressions are still the most common, but there are more runners showing expressions of anger, happiness, or even fear (see Fig. 10). Finally, RP5 has the smallest collection of individuals, with few runners in the first half of the recording time. During the second half, the angry expressions rise for a while, reflecting those runners making a final effort. However, the last runners just want to finish the race, making the neutral and sad expressions dominate again.

### Fatigue-AU trade-off

Action Units are indicators of specific facial muscle movements. Recently, Uchida et al. [35] have established a correlation between facial expressions and the intensity of sporting activity. In particular, they found that the activation frequency of the zygomatic major muscle (coded as AU12 in FACS) is remarkable for athletes undertaking a great effort. This muscle triggers the lip corner pull movement, related to physical tension and fatigue expressions.

The Py-Feat toolbox tool provides, for each analyzed face, whether a given AU has been activated or not. In order to estimate the general tendency in each RP, we have computed the percentage of detected faces with the AU12 activated. The higher this percentage, the higher the level of fatigue of the captured runners.

**Fig. 12:**
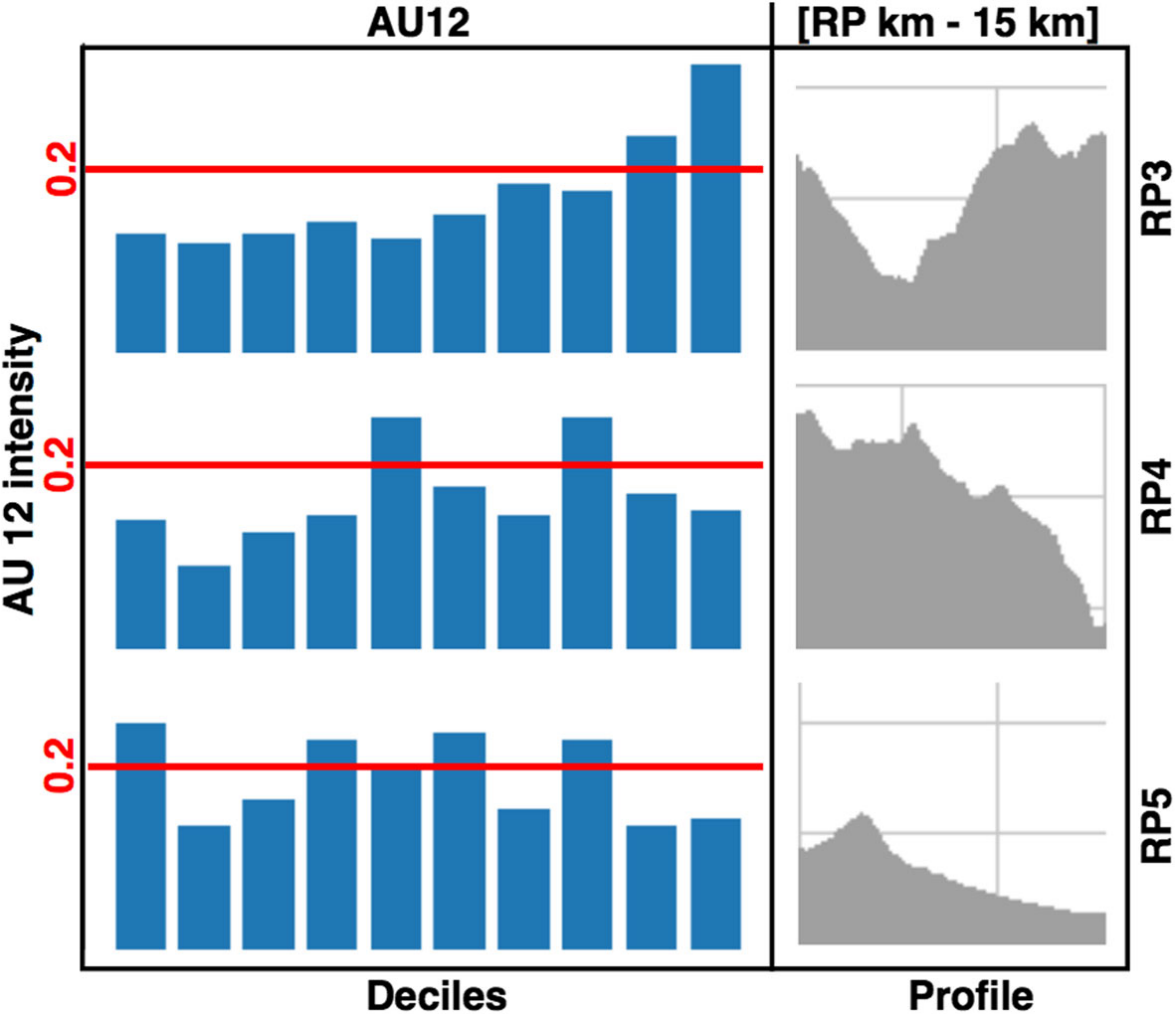
AU12 analysis. The left column shows the percentage of faces activating AU12. Data are divided chronologically by deciles. The right column shows the profile of the last 15 kilometers of the race course before reaching the corresponding RP

Figure 12 displays the results obtained. The left column shows the AU12 activation percentage divided by deciles according to the elapsed recording time. The race leaders are in the first decile (from the start of the recording to 10% of the elapsed time), while the slowest runners are in the last decile (from 90% of the elapsed time to the end of the recording). To provide further insight into the fatigue level of the runners, the right column shows the race’s profile over the 15 kilometers preceding the RP where the data were collected.

According to Uchida et al. findings, the information provided by AU12 is more relevant when athletes are tired. For this reason, we have analyzed their AU12 activation frequency after they have already run almost 100 km (RP3) from the starting line. The top graph in Fig. 12 shows the mean response of runners at this location. As can be appreciated, the AU12 activation frequency increased along with poor performance. A significant slope can be seen at the profile provided in the corresponding right column graph. This effect may suggest that the slowest runners have some difficulties with muscular fatigue.

The profile before the RP4 location shows another slope but in an opposite sense. In this case, the runners must go downhill. After running 110 kilometers, some expertise is required to address this situation adequately. This is not an issue for the race leaders, however, the middle classified runners experienced some difficulties. Moreover, these runners were aware that they were facing the last 20 kilometers of the race, and they must increase their performance to achieve a better position, affecting their running style. The last runners exhibited a conservative running style due to the hopeless chances of winning the race.

Finally, the profile before RP5 is relatively plain. Leaders exhibit a higher AU12 activation frequency due to their effort to increase their winning chances. Other runners may activate their performance to expend all of their energy before the finish line and achieve their best personal qualification time. As has happened in RP4, the last runners exhibited a conservative running style to reach the finish line.

### Fatigue indicators and individual performance

The analysis of the AU12 time distribution makes possible to assess each runner’s performance concerning the rest of the participants. However, it does not provide information about their individual performance. Ultra-distance runners are dedicated athletes who spend most of the year in hard training and often participate repeatedly in this type of event, perfectly aware of their performance during the race.

To estimate the personal performance of each runner, we have studied the rankings of previous editions of the race. The 2020 edition of the TGC ran on a similar course to the 2018 and 2019 editions, so we have checked which runners annotated in 2020 participated in the two previous editions and compared their passing times through each checkpoint. For each RP, we consider the runners are performing better if they have improved their best passing time in the previous two editions, and otherwise, we consider they are performing worse.

The routes of the three considered editions of the TGC are similar but not identical. Table 5 illustrates that, while the distance covered from the starting line to each RP does not vary much between the three years, the accumulated elevation gain shows some difference, which indicates that the route has not been the same. Hence, the purpose of this indicator is not to measure the actual performance of the runners but rather their psychological perception of their performance when reaching a particular checkpoint, and the impact of this psychological perception on their facial expression.

**Table 5:** Details of the TGC course

	Distance to			Elevation gain to		
	RP3	RP4	RP5	RP3	RP4	RP5
**2018**	82.2km	110.6km	124.7km	5652m	6645m	6887m
**2019**	82.5km	111.1km	125.2km	5652m	6639m	6881m
**2020**	82.0km	110.4km	124.4km	6146m	7267m	7607m

**Fig. 13:**
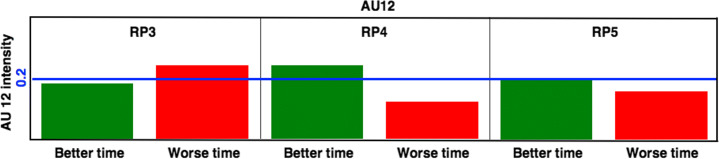
AU12 activation percentage according to runner performance. The green bar shows the AU12 activation percentage for those runners performing better in 2020 than in the two previous years, while the red bar shows the AU12 activation percentage for those runners performing worse

Figure 13 shows the AU12 activation percentage for the best and worst-performing runners in each RP. The sample is relatively small, as it only includes those runners annotated in 2020 for whom there are records in 2018 and 2019. There are 31 runners in RP3, with 20 having better performance and 11 having worse performance. There are 27 runners in RP4 (18 vs. 9) and 24 runners in RP5 (14 vs. 10). Nonetheless, these data provide an interesting insight into the relationship between runner fatigue and AU12 activation.

The AU12 activation percentage is lower for those runners performing better than in previous years in RP3 than for those performing worse. Physical fatigue is evident among those runners with poorer performance after the tough climb leading to RP3, mainly because they are still struggling to push their pace and meet the expectations they have set for themselves.

The trend is the opposite on RP4, where the runners arrive after a highly technical descent. After this tricky slope, which requires extreme concentration, the best performers show mental exhaustion on their faces. At such a late point in the race, the worst performers have lost their expectations and assumed they would be making a worse time than the previous years, so they probably reduced their rhythm to avoid injuries and save their strength for future races.

The difference between the best and worst performers is more negligible in RP5 due to the proximity to the finish line and the fact that it is a flat and easy course. Over such a long distance, runners cannot afford a final sprint, so this last stretch has little effect on their final race time. In fact, in the analyzed 2020 TGC edition, the two runners who reached the last stretch of the race with a chance of winning decided not to compete in the final meters and share the victory.

## Conclusions

This paper presents a novel analysis to determine the correlation between sports performance and facial expressions. The reported experiments prove that facial expressions play a key role in assessing runner performance. Our findings have demonstrated that the relationship between the signs of fatigue and the performance of each runner varies throughout the entire competition.

Our results also show the great difficulty of applying FER models in a wild environment. The different models provide consistent results, with emotions related to physical effort and fatigue being dominant, but they do not necessarily agree on a particular emotion. Without a doubt, we are dealing with a highly complex matter that needs to be interpreted according to the race profile, the covered distance, and the time remaining to reach the finish goal.

This line of research offers many interesting opportunities within the field of competitive sports. Among the most relevant uses, we can highlight non-invasive performance analysis applications which can provide the coaching staff with useful feedback to direct the efforts of their athletes and correct negative trends. Furthermore, monitoring the facial expression of professional athletes may provide health cues that would advert competition organizers of unpleasant situations for the participants.

## Data Availability

Data sharing not applicable to this article as no datasets were generated or analyzed during the current study.

## Footnotes

1 https://www.kaggle.com/c/challenges-in-representation-learning-facial-expression-recognition-challenge
